# Pancoast Syndrome and Middle Ear Mass as the First Manifestation of Small‐Cell Lung Carcinoma: A Case Report

**DOI:** 10.1002/ccr3.72333

**Published:** 2026-05-10

**Authors:** Motahhareh Karimoddini, Zahra Behrooznia, Amir Baniasad, Shahabaddin Sorouri, Fatemeh Amirian

**Affiliations:** ^1^ Internal Medicine Department, Faculty of Medicine Mashhad University of Medical Sciences Mashhad Iran; ^2^ Lung Disease Research Center Mashhad University of Medical Sciences Mashhad Iran

**Keywords:** Horner's syndrome, non‐small cell lung cancer (NSCLC), Pancoast syndrome, small cell lung carcinoma (SCLC)

## Abstract

Small‐cell lung carcinoma (SCLC) is a rare cause of Pancoast syndrome. We present a 54‐year‐old man with shoulder pain, arm weakness, and a middle ear mass, leading to a diagnosis of SCLC at the lung apex. This case highlights the importance of considering SCLC in the differential diagnosis of Pancoast syndrome. Early recognition and a comprehensive diagnostic approach are crucial for optimizing patient outcomes.

## 1 Introduction

Pancoast syndrome is a rare clinical condition characterized by a triad of symptoms: shoulder pain, Horner's syndrome, and weakness in the upper extremities [1]. Horner syndrome is an interruption in the oculosympathetic pathway, resulting in ptosis, miosis, and, less commonly, anhidrosis of the brow and face [2]. Pancoast syndrome is typically caused by apical lung tumors, commonly referred to as Pancoast tumors, which are most often associated with non‐small cell lung cancer (NSCLC), squamous cell carcinoma, or adenocarcinoma [3]. However, Pancoast syndrome can also occur as an early manifestation of small cell lung carcinoma (SCLC), although this presentation is exceedingly rare [4]. SCLC is an aggressive type of cancer characterized by rapid growth, early metastasis, and a strong association with smoking [5].

This case report describes a rare instance of Pancoast syndrome presenting as the initial clinical manifestation of SCLC, highlighting the diagnostic challenges and complexities involved in its management. It underscores the critical importance of recognizing atypical presentations of SCLC, as timely diagnosis and intervention can significantly improve patient outcomes.

## 2 Case History and Physical Examinations

### 2.1 History/Examination

A case of a 54‐year‐old male farmer and chronic smoker (33 pack years) presented with severe pain in the left shoulder, limited range of motion, significant weight loss of 20 kg in 2 months, night sweating, productive cough, and dyspnea on exertion classified as New York Heart Association (NYHA) class II. Gradually, the patient developed ptosis, anhidrosis on the left side, purulent otorrhea on the left side, and hoarseness of voice. Upon examination, the patient was conscious, and his vital signs were stable. He had no fever. Lung auscultation revealed decreased breath sounds in the apex of the left lung. Heart auscultation findings were normal. There was no lymphadenopathy. The left shoulder exhibited a limited range of motion in all directions during active movements. Still, passive movements were painful despite a normal range of motion, and the muscles were not atrophied. The force of the left upper limb was 3/5 in the proximal part and 5/5 in the distal part. Ptosis and miosis with mild peripheral facial paresis were evident on the left side. Microscopic examination of the left ear revealed purulent otorrhea with a soft tissue mass in the middle ear.

### 2.2 Investigations, Diagnosis and Treatment

**FIGURE 1:**
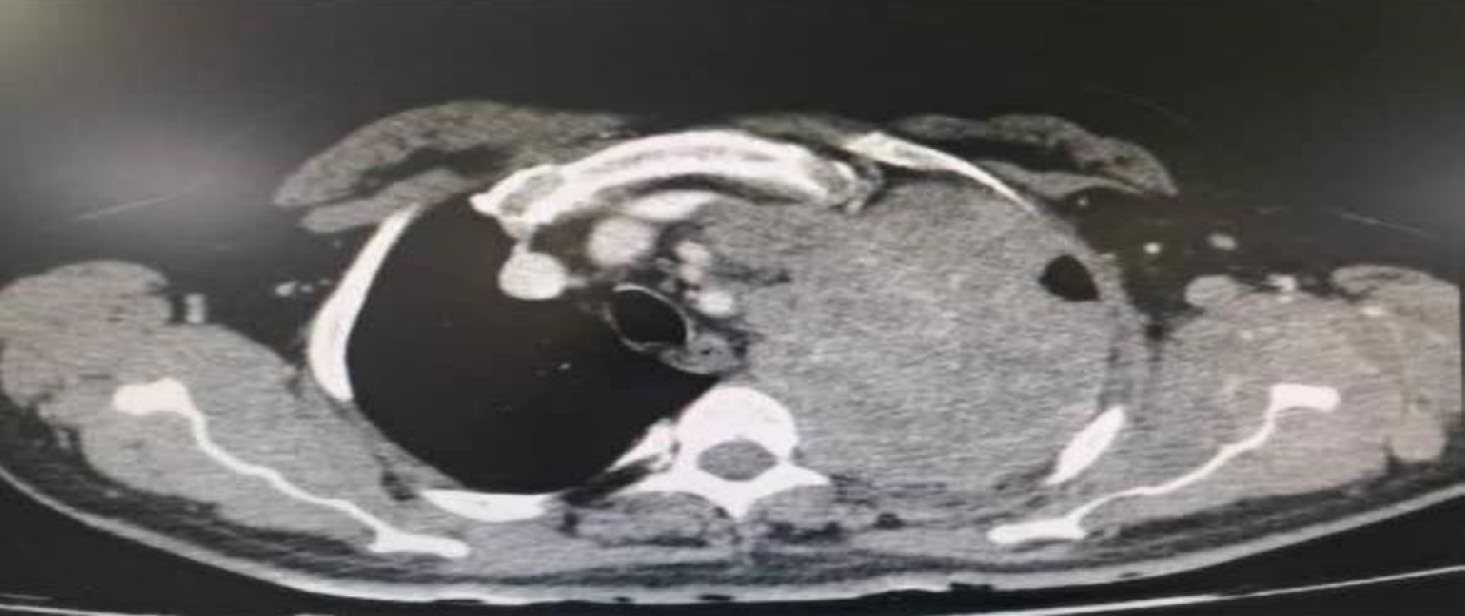
A large mass measuring 114 × 82 mm in the left upper lobe extends into the anterior and middle mediastinum. It encases the left main pulmonary branch, lobar, and segmental branches of both the upper lobe and lingula.

**FIGURE 2:**
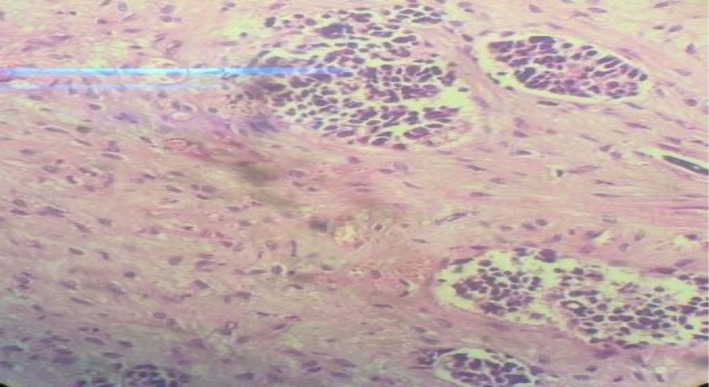
Sections of left middle ear mass biopsy show nondysplastic stratified squamous epithelium, sub‐tissue with a nest of neoplastic proliferation of atypical cells with hyperchromatic nuclei, high nuclear‐cytoplasmic ratio, and crush artifact, compatible with small round blue cell tumor. (H&E staining with 40× magnification).

**FIGURE 3:**
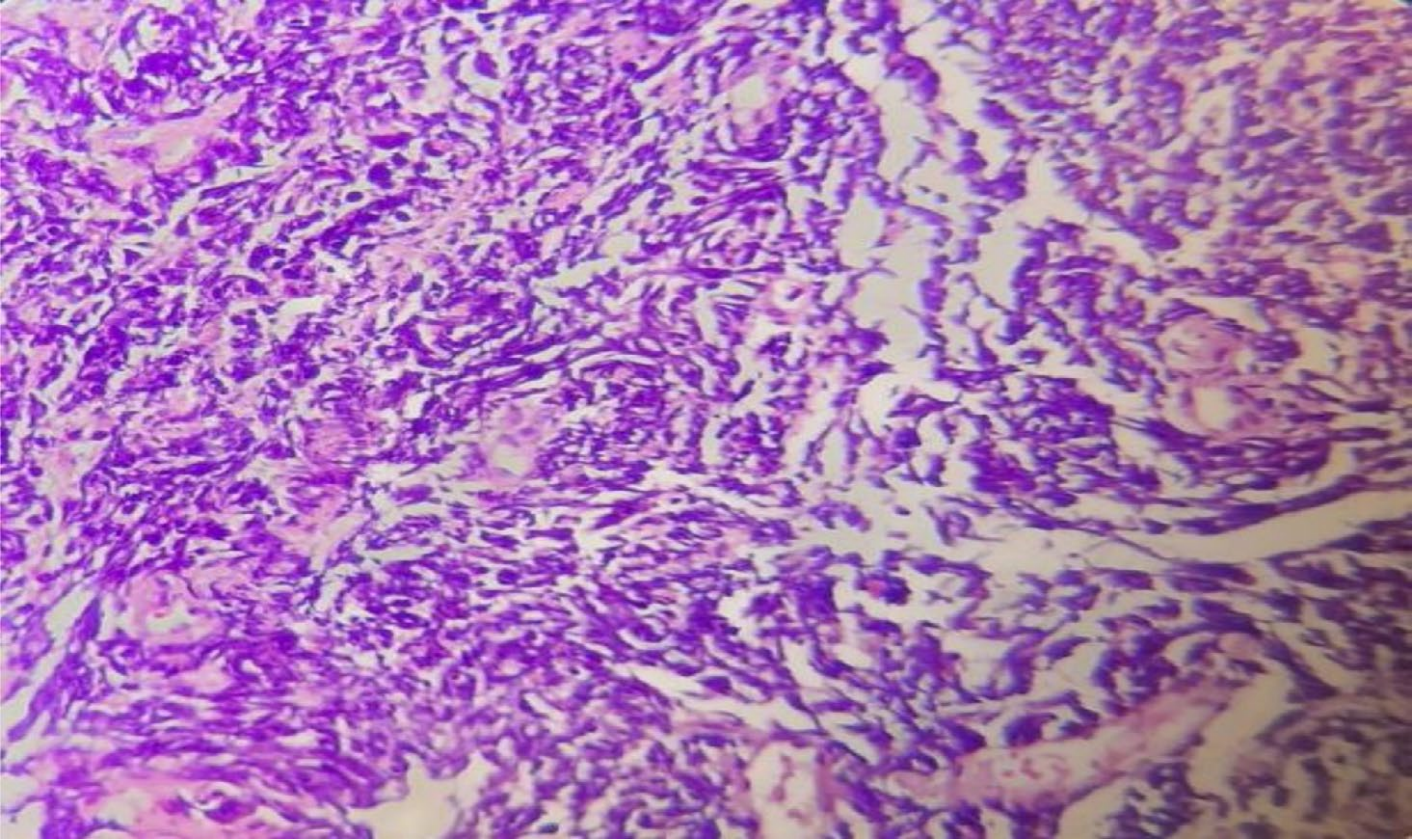
Bronchial biopsy sections show respiratory mucosa involved by a carcinoma consisting of sheets of atypical cells with a high nuclear‐cytoplasmic ratio and hyperchromatic nuclei, with an extensive crushed artifact compatible with small cell carcinoma (H&E staining with 40× magnification).

Laboratory tests showed CRP: 19.2 mg/L (normal range: > 6) and LDH: 2036 U/L (normal range: 100–480), while other laboratory tests were normal. The lung computed tomography (CT) scan revealed a large mass measuring 114 × 82 mm in the left upper lobe. The mass shows infiltration extending into the anterior and middle mediastinum, encasing the left main pulmonary branch and the lobar and segmental branches of both the upper lobe and lingula (Figure 1). The brain CT scan was normal. According to the otorhinolaryngology consultation, ear secretions were suctioned, along with a biopsy of the middle ear mass, which revealed a small, round, blue cell tumor (Figure 2). A bronchial mass biopsy also indicated bronchial small cell carcinoma (Figure 3). Immunohistochemistry (IHC) testing was performed to determine the origin of a small, round, blue cell tumor. The test indicated that Thyroid transcription factor 1 (TTF1) was positive, confirming that the tumor originated in the lung.

### 2.3 Outcome and Follow‐Up

The patient underwent chemotherapy administered by a hematology oncologist. After receiving two courses of chemotherapy, the patient's symptoms improved. The patient is currently undergoing chemotherapy.

## 3 Discussion

Pancoast tumors are usually associated with NSCLC, and only rare cases of small cell carcinoma present with Pancoast syndrome [6]. SCLC accounts for 15% of all lung cancers. It is known for its rapid growth, early metastasis, and association with paraneoplastic syndromes [7, 8]. Pancoast tumors are generally slow‐growing NSCLCs that gradually invade adjacent structures over time [9].

However, in this patient, small‐cell carcinoma localized to the apex of the lung presented with classic Pancoast symptoms, including Horner's syndrome, shoulder pain, and involvement of adjacent structures such as cranial nerves and possibly the middle ear [10, 11]. The involvement of the middle ear and the subsequent otorrhea raises important considerations regarding the local spread of the tumor. While direct spread to the middle ear is uncommon, it may indicate the dissemination of malignancy to adjacent structures via the Eustachian tube [12]. Chronic smoking history plays a key role in the development of small‐cell carcinoma, as smoking is a known risk factor for all types of lung cancer, including SCLC [13, 14]. As seen in this case, despite being aggressive and having a poor prognosis, small‐cell carcinoma can respond well to primary chemotherapy [15, 16].

David H. Johnson and colleagues reported a case involving a 52‐year‐old female smoker who experienced persistent and severe pain in her left upper chest and axilla for 8 months. This pain worsened over time, eventually radiating to her left shoulder and down her medial arm to the elbow. It was linked to a left upper lobe lung mass and SCLC, which are rare manifestations of a Pancoast tumor [17]. Jefferson Fontinele e Silva and colleagues reported a 74‐year‐old smoker with brachial plexus involvement and rib and vertebral erosions. Notably, the patient did not show signs of Horner's syndrome but experienced a rapid onset of neurological symptoms and early indications of metastasis. This presentation suggests that small cell carcinoma is more aggressive than NSCLC [18]. A similar case was reported by Sion Hangma Limbu et al., in which a 71‐year‐old patient with a history of chronic smoking developed Pancoast syndrome secondary to SCLC. The patient described in this report presented with significant weight loss, Horner's syndrome, and shoulder pain. This mirrors the case discussed, highlighting an aggressive clinical course of SCLC with local spread to nearby neurovascular structures. Despite the severity of these cases, they were generally well tolerated and responded to primary chemotherapy. The distinctive manifestation of small cell carcinoma as Pancoast syndrome arises from the tumor's location in the superior pulmonary sulcus [19, 20]. Tumors in this region can invade the brachial plexus, the cervical sympathetic chain, the subclavian vessels, and the vertebral bodies. This invasion may result in the characteristic symptoms of Pancoast syndrome, which include severe shoulder pain, Horner's syndrome, and, in some cases, neurological deficits in the upper extremities [3, 21].

In the case presented, the absence of muscle atrophy and shoulder pain indicates early brachial plexus involvement, which may lead to a better treatment response. This contrasts with more advanced Pancoast tumors associated with NSCLC, where patients often experience significant muscle atrophy and irreversible nerve damage by the time the diagnosis is made [1, 22]. Although small‐cell lung carcinoma typically exhibits aggressive characteristics, it can, in rare cases, remain localized. This localized form of the disease allows for effective symptom management with chemotherapy, despite the generally poor long‐term prognosis due to a high likelihood of recurrence and metastasis [23]. In the study by D. M. Spengler et al., musculoskeletal complaints were the presenting symptoms in about 90% of patients with Pancoast syndrome [24]. In our patients, left shoulder pain and limited range of motion were among the presenting symptoms. So, orthopedists, who may be the first to encounter patients later diagnosed with this condition, must be aware of its signs and symptoms.

## 4 Conclusion

This case underscores the importance of recognizing Pancoast syndrome as a potential manifestation of small cell carcinoma despite its rarity. Early detection of these symptoms, along with appropriate diagnostic procedures such as imaging and biopsy, is crucial for guiding treatment. In this instance, the patient experienced clinical improvement due to the early initiation of chemotherapy. Ongoing surveillance will be essential, given the high recurrence rate of small cell carcinoma. Further studies are needed to investigate how Pancoast syndrome presents in small cell carcinoma and to understand its possible implications for treatment outcomes.

## Author Contributions

**Motahhareh Karimoddini:** conceptualization, data curation, investigation, methodology, resources, software, writing – original draft, writing – review and editing. **Zahra Behrooznia:** conceptualization, project administration, visualization. **Amir Baniasad:** investigation, visualization. **Shahabaddin Sorouri:** conceptualization, data curation, investigation, methodology, resources, software, writing – original draft, writing – review and editing. **Fatemeh Amirian:** project administration, visualization.

## Funding

The authors have nothing to report.

## Consent

Written informed consent was obtained from the patient to publish this report in accordance with the journal's patient consent policy.

## Conflicts of Interest

The authors declare no conflicts of interest.

## Data Availability

The data that support the findings of this study are available from the corresponding author upon reasonable request.

## References

[1] L. de Oliveira Cardoso , S. R. Guedes , L. Barbosa Fiúza , et al., “Clinical Manifestations and Repercussions of Pancoast Tumor,” Brazilian Journal of Implantology and Health Sciences 6, no. 10 (2024): 4589–4602.

[2] T. J. Martin , “Horner Syndrome: A Clinical Review,” ACS Chemical Neuroscience 9, no. 2 (2018): 177–186. 29260849 10.1021/acschemneuro.7b00405

[3] E. T. Darmadi and I. Tjokrowinoto , “Pancoast Tumor: A Case Report,” Jurnal Widya Medika 8, no. 2 (2022): 181–202.

[4] N. Panagopoulos , V. Leivaditis , E. Koletsis , et al., “Pancoast Tumors: Characteristics and Preoperative Assessment,” Journal of Thoracic Disease 6, no. Suppl 1 (2014): S108–S115. 24672686 10.3978/j.issn.2072-1439.2013.12.29 PMC3966151

[5] Q. Wang , Z. H. Gümüş , C. Colarossi , et al., “SCLC: Epidemiology, Risk Factors, Genetic Susceptibility, Molecular Pathology, Screening, and Early Detection,” Journal of Thoracic Oncology 18, no. 1 (2023): 31–46. 36243387 10.1016/j.jtho.2022.10.002 PMC10797993

[6] O. Elsaka , M. A. Noureldean , M. A. Gamil , M. T. Ghazali , A. H. Abd Al‐Razik , and D. Hisham , “Pathophysiology, Investigations, and Management in Cases of Pancoast Tumor,” Asian Research Journal of Current Science 4 (2022): 83–100.

[7] C. M. Rudin , E. Brambilla , C. Faivre‐Finn , and J. Sage , “Small‐Cell Lung Cancer,” Nature Reviews Disease Primers 7, no. 1 (2021): 3. 10.1038/s41572-020-00235-0 PMC8177722 33446664

[8] M. S. Alam , G. Malik , P. Tanwar , et al., “A Review on Small‐Cell Lung Cancer: Epidemiology, Pathophysiology, RiskFactors, Diagnosis, Clinical Management and Treatment Modalities,” International Journal of Current Science Research and Review (IJCSRR) 6, no. 1 (1969): 129–151.

[9] A. Çinkooğlu and R. Savaş , “Imaging of Lung Cancer,” in Airway Diseases (Springer, 2023), 1053–1091.

[10] C. Thompson , R. Daley , S. Fullerton , S. Lambert‐Johnson , and D. K. Johnson , “Shoulder and Arm Pain: A “Red Herring” Chief Complaint in a Smoker Diagnosed With Pancoast‐Tobías Syndrome,” Journal of Case Reports and Images in Oncology 10, no. 1 (2024): 1–6.

[11] F. Handayani and F. P. Sinambela , “Impact of Chemotherapy on Renal Parameters and Treatment Outcomes in Nasopharyngeal Carcinoma Patients: A Retrospective Study at Santa Elisabeth Hospital Batam,” International Journal on ObGyn and Health Sciences 2, no. 1 (2023): 1–8.

[12] B. L. Njaa , “Tumors of the Ear,” in Tumors in Domestic Animals, ed. D. J. Meuten (Wiley Blackwell, 2016), 923–941.

[13] N. Rekhtman , S. E. Tischfield , C. A. Febres‐Aldana , et al., “Chromothripsis‐Mediated Small Cell Lung Carcinoma,” Cancer Discovery 15, no. 1 (2025): 83–104. 39185963 10.1158/2159-8290.CD-24-0286 PMC11726019

[14] R. Dorantes‐Heredia , J. M. Ruiz‐Morales , and F. Cano‐García , “Histopathological Transformation to Small‐Cell Lung Carcinoma in Non‐Small Cell Lung Carcinoma Tumors,” Translational Lung Cancer Research 5, no. 4 (2016): 401–412. 27652204 10.21037/tlcr.2016.07.10 PMC5009079

[15] D. Cortinovis , P. Bidoli , S. Canova , et al., “Novel Cytotoxic Chemotherapies in Small Cell Lung Carcinoma,” Cancers 13, no. 5 (2021): 1152. 33800236 10.3390/cancers13051152 PMC7962524

[16] W. J. Petty and L. Paz‐Ares , “Emerging Strategies for the Treatment of Small Cell Lung Cancer: A Review,” JAMA Oncology 9, no. 3 (2023): 419–429. 36520421 10.1001/jamaoncol.2022.5631

[17] P. Thaisetthawatkul and P. J. B. Dyck , “Cervical and Lumbosacral Radiculoplexus Neuropathies,” in Dysimmune Neuropathies (Elsevier, 2020), 199–223.

[18] J. Fontinele e Silva , P. Barbosa Mde , and C. L. Viegas , “Small Cell Carcinoma in Pancoast Syndrome,” Jornal Brasileiro de Pneumologia 35, no. 2 (2009): 190–193. 19287924 10.1590/s0101-28002009000300004

[19] S. H. Limbu , N. Bhatta , D. R. Mishra , et al., “Small Cell Lung Carcinoma With Pancoast Syndrome: A Case Report,” JNMA; Journal of the Nepal Medical Association 60, no. 246 (2022): 211–213. 35210644 10.31729/jnma.6620 PMC9199995

[20] C. Almanzar , S. Maxwell , M. Gomez , O. Ansari , and L. Silva , “Pancoast Syndrome due to High Grade Anaplastic Tumor,” HCA Healthcare Journal of Medicine 2, no. 1 (2021): 35. 37424884 10.36518/2689-0216.1076 PMC10324725

[21] J. M. S. Pérez , “Cervicobraquialgia y Ptosis Palpebral Como Manifestación de Tumor Pulmonar,” Medicina General 7, no. 5 (2018): 9.

[22] K. McLaughlin , K. S. Tan , J. Dycoco , et al., “Superior Sulcus Non–Small Cell Lung Cancers (Pancoast Tumors): Current Outcomes After Multidisciplinary Management,” Journal of Thoracic and Cardiovascular Surgery 166, no. 6 (2023): 1477–1487.e8. 37611845 10.1016/j.jtcvs.2023.08.023 PMC11229055

[23] Z. Megyesfalvi , C. M. Gay , H. Popper , et al., “Clinical Insights Into Small Cell Lung Cancer: Tumor Heterogeneity, Diagnosis, Therapy, and Future Directions,” CA: A Cancer Journal for Clinicians 73, no. 6 (2023): 620–652. 37329269 10.3322/caac.21785

[24] D. M. Spengler , M. M. Kirsh , and H. Kaufer , “Orthopaedic Aspects and Early Diagnosis of Superior Sulcus Tumor of Lung (Pancoast),” Journal of Bone and Joint Surgery. American Volume 55, no. 8 (1973): 1645–1650. 4804986

